# Sustained Effectiveness of the Maternal Pertussis Immunization Program in England 3 Years Following Introduction

**DOI:** 10.1093/cid/ciw559

**Published:** 2016-11-02

**Authors:** Gayatri Amirthalingam, Helen Campbell, Sonia Ribeiro, Norman K. Fry, Mary Ramsay, Elizabeth Miller, Nick Andrews

**Affiliations:** 1 Immunisation, Hepatitis and Blood Safety Department; 2 Respiratory and Vaccine Preventable Bacterial Reference Unit; 3 Statistics, Modelling and Economics Department, Public Health England, London, United Kingdom

**Keywords:** maternal pertussis vaccination, vaccine effectiveness

## Abstract

The effectiveness of maternal immunization in preventing infant pertussis was first demonstrated in England, 1 year after the program using diphtheria–tetanus–5-component acellular pertussis–inactivated polio vaccine (dT5aP-IPV) was introduced in 2012. Vaccine effectiveness against laboratory-confirmed pertussis has been sustained >90% in the 3 years following its introduction, despite changing to another acellular vaccine with different antigen composition. Consistent with this, disease incidence in infants <3 months of age has remained low despite high activity persisting in those aged 1 year and older. Vaccine effectiveness against infant deaths was estimated at 95% (95% confidence interval, 79%–100%). Additional protection from maternal immunization is retained in infants who received their first dose of the primary series. There is no longer evidence of additional protection from maternal vaccination after the third infant dose. Although numbers are small and ongoing assessment is required, there is no evidence of increased risk of disease after primary immunization in infants whose mothers received maternal vaccination.

Pertussis resurgences have been reported in recent years from a number of countries with longstanding vaccination programs. Global efforts have therefore focused on better understanding the underlying reasons for the increases and consideration of strategies for optimizing protection of young infants, who are at highest risk of severe disease and death. In the 2015 World Health Organization (WHO) pertussis position paper, the evidence for a range of additional strategies including cocooning and maternal immunization were reviewed, drawing from the experience of countries in which these strategies had been implemented with varying degrees of success [1].

Maternal immunization has been recommended in a number of countries including the United Kingdom, the United States, Australia, Argentina, Belgium, and Spain [2–7]. In the United Kingdom, an emergency program to offer pertussis vaccination to pregnant women was introduced in October 2012, in response to a rise in hospitalizations and deaths among unimmunized infants <3 months of age, with 14 pertussis-related infant deaths reported in the 2012 peak [8]. Many other countries have also observed an increasing burden of infant disease, with an average of 3055 infant pertussis cases and >19 infant deaths reported each year in the United States since 2004 [9]. The recommendation to offer a low-dose diphtheria–tetanus–5-component acellular pertussis–inactivated polio combination vaccine (dT5aP-IPV) to women in every pregnancy, ideally between 28 and 32 weeks, was considered the best way to protect infants from birth, which is through passive immunization from transplacentally derived maternal antibodies. The United Kingdom was the first country to demonstrate high levels of protection against disease in infants from maternal immunization using 2 different methodological approaches. Vaccine effectiveness in the first year of the program was estimated to be >90% for infants <2 months of age, whose mothers received dT5aP-IPV at least 1 week prior to delivery [10, 11].This was further supported by ecological evidence of a direct impact on laboratory-confirmed reports and hospitalizations in the target age group [10]. The safety of the maternal pertussis immunization program has also been supported by the findings of a large observational study in the United Kingdom, which did not identify an increased risk for a range of maternal, fetal, and neonatal outcomes among almost 18 000 vaccinated pregnant women and their infants [12]. In 2014, in the face of continuing raised levels of pertussis activity in the population and good evidence supporting the safety and effectiveness of the program, the national advisory committee, the Joint Committee on Vaccination and Immunisation (JCVI), advised that this outbreak response measure should continue for a further 5 years [13]. In the United Kingdom, vaccines for the national program are centrally procured and distributed and in July 2014, the vaccine product being offered to pregnant women changed from dT5aP-IPV (pertussis toxin [PT]: 2.5 µg; filamentous hemagglutinin [FHA]: 5 µg; pertactin [PRN]: 3 µg; fimbriae types 2 and 3 [FIM]: 5 µg) to diphtheria–tetanus–3-component acellular pertussis (dT3aP-IPV) (PT: 8 µg; FHA: 8 µg; PRN: 2.5 µg).

Despite the reassuring data on safety and effectiveness of maternal immunization, one of the key remaining questions has been the impact of interference from maternally derived antibodies on the infants' immune response and the resultant risk of disease among older fully vaccinated infants and toddlers. While a number of published studies from the United Kingdom, Belgium, and North America have demonstrated blunting of pertussis responses and other antigens in the routine program in infants born to vaccinated mothers, the clinical significance of these effects remains unknown due to a lack of any agreed correlates of protection for pertussis [14–17]. In this article, we update the previously published estimates of vaccine effectiveness (VE) of the maternal program in England, 3 years following its introduction, and provide the first estimates for the effectiveness of the maternal program on infant deaths and the comparative effectiveness of dT5aP-IPV and dT3aP-IPV on infant disease. An early assessment of the potential impact of blunting on clinical disease is also presented.

## METHODS

### Data Sources

#### Vaccine Coverage

In England, the delivery of the maternal pertussis vaccination program predominantly occurs in general practice and, therefore, primary care data provide the most accurate and reliable source of coverage data. In England, this can be derived from 2 primary care data sets. The first source is the routine collection (Immform), which measures coverage at national and subnational levels on a monthly basis using data held on computerized general practice records. Since April 2014, this has moved from a manual to an automated extraction with participation of >90% of general practices in England. In each survey month, the denominator is the number of pregnant women with an estimated date of delivery in that month and the numerator is the number of women who received pertussis vaccine after 28 weeks of gestation.

The second data source is a sentinel primary care data source, the Clinical Practice Research Datalink (CPRD), which represents approximately 6% of the UK population and includes 520 English general practices. The CPRD has been used to evaluate the maternal program in England and the methods previously described [10]. Data from the CPRD were extracted in November 2015 and coverage was calculated by week of the child's birth for the period 1 October 2012 to 31 August 2015. The cohort was defined as any woman with a READ code for a live birth from 1 October 2012. For each week, the denominator was the number of women from participating practices that delivered a live infant in that week; the numerator was the number of women who received a pertussis vaccine during pregnancy. Only pertussis vaccines recorded as administered between 300 days prior to a birth and up to 8 weeks after birth were counted. The additional benefit of the CPRD over the routine collection is that the precise timing of vaccination during pregnancy can be derived. To determine likely vaccine product administered, 1 July 2014 was used as the switch from dT5aP-IPV to dT3aP-IPV. As data are analyzed according to the child's date of birth, there is a period during which children born may have had mothers vaccinated by either vaccine.

#### Laboratory-Confirmed Pertussis

In England, Public Health England (PHE) is responsible for the national surveillance of vaccine-preventable infections with detailed individual follow-up of all laboratory-confirmed pertussis cases. Laboratory confirmation can either occur at the local hospital microbiology laboratory (culture); through the regional PHE laboratory network (polymerase chain reaction available through the regional network since July 2014 for all age groups, previously only offered for hospitalized infants by the national reference laboratory); or at the national reference laboratory (serological testing available since 2001 for all age groups and, since January 2013, oral fluid testing for suspected cases initially aged 8–16 years and extended to those aged 5–16 years from July 2013). Both serology and oral fluid testing are based on demonstrating high anti-PT immunoglobulin G titers above a predefined threshold considered indication of recent infection and may be confounded by pertussis vaccination within the previous year [18].

All laboratory-confirmed cases are followed up with the patients' general practitioner to collect additional clinical and epidemiological data including vaccination history and, for infants born after 1 October 2012, the maternal vaccination status. Since July 2014, with changes to the vaccine product being offered through the national program, additional information on the vaccines administered is collected for both the childhood course and maternal vaccination where applicable.

#### Pertussis-Related Deaths

Pertussis deaths are reconciled from the following data sources: registered deaths from the Office of National Statistics; deaths identified from the routine follow-up of laboratory-confirmed reports; and those identified in the Hospital Episode Statistics dataset and on HPZone (InFact UK Ltd), a secure Web-based information system used to record local public health management of individual cases of infectious diseases.

### Statistical Analysis

#### Maternal Vaccine Effectiveness Against Infant Disease and Death

Vaccine effectiveness against laboratory-confirmed infant disease was calculated using the screening method where VE is 1 minus the odds of vaccination in cases (maternal vaccine status) divided by the odds of vaccination in the matched population. Statistically this was done by logistic regression with an offset for the logit of the matched population coverage. Detailed methods have been previously described [10, 19].

For the analysis, the expected coverage in the mother was determined for each confirmed case using the CPRD dataset matched on the week of birth of the baby and the birth cohort of the mother (pre-1985, 1985–1989, 1990 and later). If mother's date of birth was unknown, the average coverage was used. For the primary analysis, when calculating expected vaccine coverage, any vaccine given within 7 days of birth was excluded from the calculation. Similarly, any cases whose mothers were vaccinated within 7 days of birth were not included. Cases were included if the age at onset was known, or otherwise if date of specimen collection was before the age of 24 months (≤731 days). VE was assessed for cases with onset/sample aged ≤62 days and cases with onset/sample aged ≤93 days as long as they had not received their first routine dose within 7 days of onset/sample.

To account for differences observed between CPRD coverage and Immform coverage, a sensitivity analysis was done with coverage reduced by a relative 20% (eg, 70% reduces to 56%) to more closely match Immform coverage.

Analysis was undertaken to calculate the following VE measures: (1) maternal VE against infant disease; (2) maternal VE by timing of vaccination; (3) maternal VE against infant death from pertussis; and (4) maternal VE against infant disease for dTaP5-IPV and dTaP3-IPV vaccines.

#### Maternal Vaccine Effectiveness in Infants Commencing Primary Infant Series

To address the question of whether maternal vaccination may have a detrimental effect on responses to primary vaccination, the VE of the maternal vaccine in children who have received primary vaccine doses was assessed. An infant dose was counted if given >7 days prior to onset/sample date. Sample date may be weeks after onset date, so children with only a sample date may in fact have had onset prior to vaccine doses. Children up to 23 months of age were included and maternal VE was assessed after the infant had received 1, 2, and 3 primary doses. An additional analysis using only cases with a known onset date was done to reduce possible misclassification of vaccination status.

## RESULTS

### Maternal Vaccine Coverage

**Figure 1.**
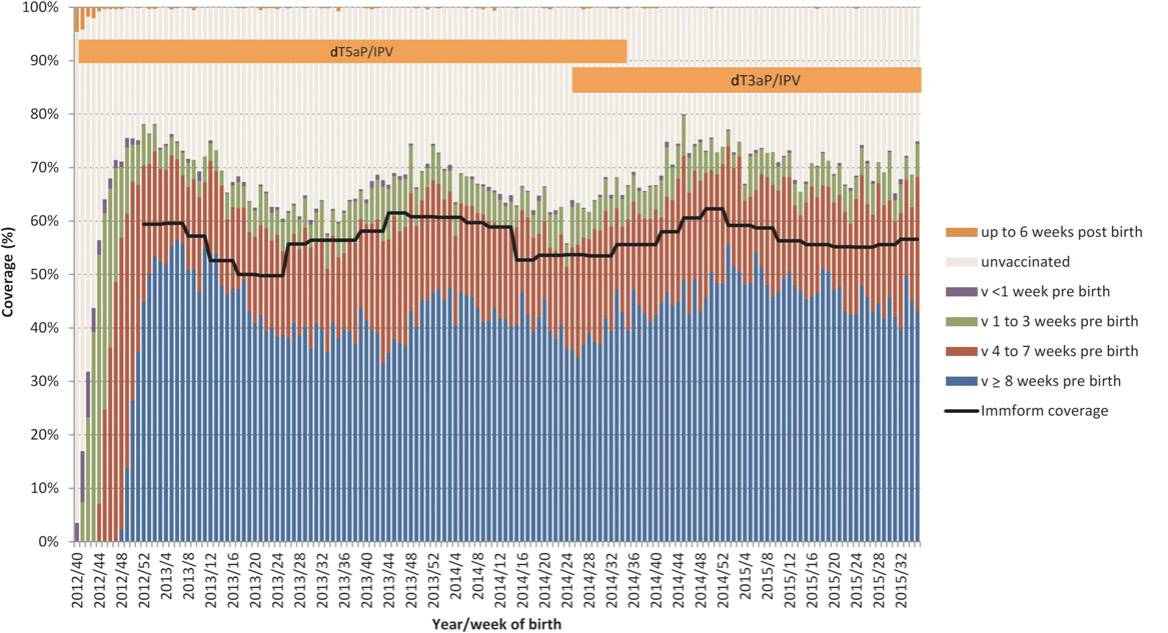
Maternal pertussis vaccine coverage, England, October 2012–August 2013, Clinical Practice Research Datalink (CPRD) and Immform. Denominator for CPRD is number of women delivering a live infant, by week of birth; denominator for Immform is number of pregnant women with an estimated date of delivery, each month. Abbreviations: dT3aP, diphtheria–tetanus–3-component acellular pertussis vaccine; dT5aP, diphtheria–tetanus–5-component acellular pertussis vaccine; IPV, inactivated polio vaccine.

The coverage of the maternal program in England achieved in the first year of the program has been sustained over the subsequent 2 years, with monthly coverage collected through the national Immform dataset indicating coverage sustained at between 50% and 62% from January 2013 to December 2015 (Figure 1). However, there does appear to be fluctuation over the year, particularly evident in 2013 and 2014, with coverage increasing during September to January and lower between April to August.

From the CPRD, a total of 72 781 live births from 1 October 2012 until 31 August 2015 were obtained. Vaccination coverage by week of birth and timing of vaccination relative to the birth is shown in the figure. Coverage reached a peak of 78% in mothers giving birth in the first week of January 2013, but has since shown fluctuations similar to the pattern observed through the routine Immform collection, with coverage higher over the winter months (70% in December 2013) and lower in the summer months (60% in those giving birth in August 2013). Coverage increased in 2015 to 70% and has remained stable to the end of August 2015.

Following introduction of the vaccine programme, the majority (more than two-thirds) of women vaccinated received vaccine at least 8 weeks prior to delivery. The figure 1 also shows which vaccine the mothers will have received and a period of uncertainty from week 27 to week 38 (July to mid-September 2014).

### Impact on Disease Epidemiology

**Figure 2.**
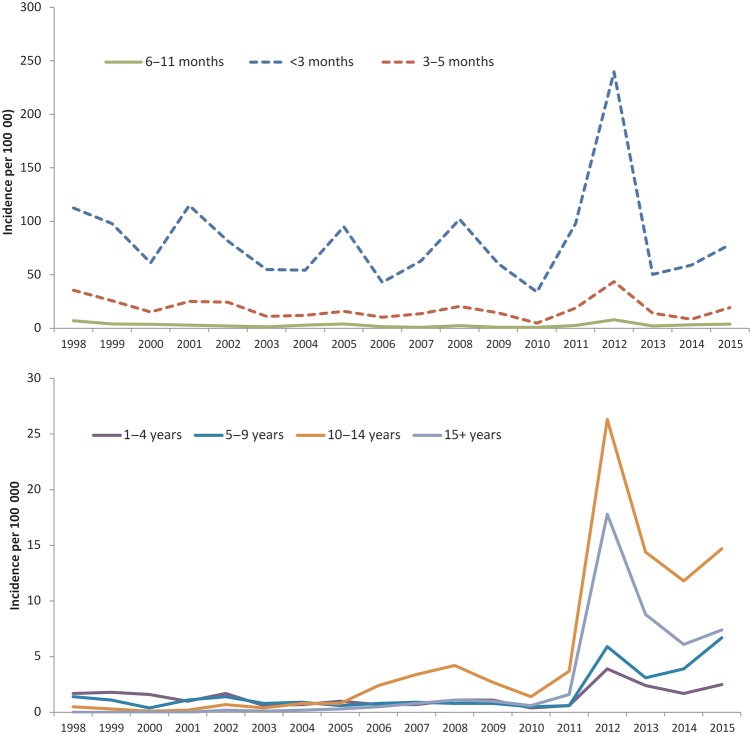
Incidence of laboratory-confirmed pertussis, by year and age group, England only, 1998–2015.

Pertussis incidence fell from a peak of 17.6 per 100 000 in 2012 to 8.6, 6.2, and 7.7 per 100 000 in 2013, 2014, and 2015, respectively. In 2015, there was an increase in all age groups relative to 2014 totals (Figure 2) and in line with expected cycles in disease incidence every 3–4 years. The average incidence of 7.5 per 100 000 in England over the 3 years following the introduction of the maternal program was 1.8 times higher than the 4.1 per 100 000 population in the 3 years preceding the program's introduction. This overall increase in incidence relative to the pre-2012 period was observed in all age groups 6 months and older (Figure 2), with the combined 3-year comparator periods (2009–2011 vs 2013–2015) increasing from 1.5 to 3.1 per 100 000 in those aged 6–11 months (2.1 times higher); 0.7 to 2.2 per 100 000 in those aged 1–4 years (3.1 times higher); 0.6 to 4.6 per 100 000 in those aged 5–9 years (7.7 times higher); 2.6 to 13.6 per 100 000 in those aged 10–14 years (5.2 times higher); and 1.1 to 7.4 per 100 000 in those aged ≥15 years (6.7 times higher). The greatest increase was observed in children aged 5–9 years of age who would have all received a complete primary and booster course of acellular pertussis–containing vaccine from mid-2013 onward. The ascertainment of cases aged 5–16 years has been enhanced with the availability of oral fluid testing from 2013: between 2013 and 2015 respectively, 14%, 21%, and 29% of cases aged 5–9 years and 14%, 15%, and 19% of those aged 10–14 years were confirmed with oral fluid.

In contrast, despite a small increase in incidence in 2015, cases in infants aged <3 months and 3–5 months were slightly lower in the most recent 3-year period (60.4 and 12.7 per 100 000, respectively) relative to the 3 years before the maternal program was introduced (62.5 and 14.0 per 100 000).

In line with this, annual deaths in infants diagnosed with pertussis have remained lower than the peak of 14 in 2012, although there has been some variation in case fatality rate in young infants over the years (Table 1). In 14 of the 16 deaths that occurred between 2013 and 2015, the infant's mother had not received pertussis vaccine in pregnancy. Only 2 of the infants born after the introduction of the maternal program had a mother who had been vaccinated during pregnancy but in both cases too close to delivery (<10 days) to confer optimal passive protection in the infant. All but 1 of the deaths since the maternal program was introduced have occurred in unimmunized infants; 1 infant had received the first dose of pertussis-containing vaccine 8 days before recorded onset, and the infant's mother, who was eligible for vaccination in pregnancy, was unimmunized.

**Table 1.** Summary of Deaths in Infant With Laboratory-Confirmed Pertussis Cases From All Sources, England Only, 2009–2015

Age at Onset	2009	2010	2011	2012	2013	2014	2015
<2 mo, unvaccinated mother	2	1	5	12	3	5	2
2–5 mo, unvaccinated mother	1	0	0	2	0	1	1
<2 mo, vaccinated mother					0	1	1
2–5 mo, vaccinated mother					0	0	0
Total deaths	3	1	5	14	3	7	4
CFR in infants <3 mo (%)	3.0%	1.8%	3.0%	3.4%	3.5%	7.1%	3.1%

Abbreviation: CFR, case fatality rate.

#### Maternal Pertussis Vaccine Effectiveness

##### Updated Estimates of Maternal Vaccine Effectiveness Against Infant Disease, Prior to Primary Immunization

By the end of September 2015, a total of 398 cases aged <24 months and born from 1 October 2012 with onset by 30 September 2014 had been confirmed (excluding serology only). Maternal vaccination status was unknown in 46 cases and so these were excluded. For this analysis, only those 253 infants aged <93 days and with no childhood doses recorded were retained. For the primary analysis of vaccination at least 7 days prior to birth, 8 individuals born in week 40 of 2012 did not contribute, as they could not have been vaccinated more than a week before birth. Two cases vaccinated within a week of birth were also excluded for this analysis. This left 243 cases, of whom 35 had been born to vaccinated mothers, giving an overall effectiveness of 91% (95% confidence interval [CI], 88%–94%) and for infants <3 months of age and 90% (95% CI, 86%–93%) for infants <2 months of age. Vaccine effectiveness remains high at 82% (95% CI, 74%–88%) for infants <2 months of age, despite reducing coverage by a relative 20% (Table 2).

**Table 2.** Maternal Pertussis Vaccine Effectiveness Estimates for Vaccinated at Least 1 Week Before Delivery

Age	Cases Vaccinated/Total	Average Matched Coverage	VE (95% CI)	VE Reducing Coverage by Relative 20% (95% CI)
<3 mo	35/243	64.8%	91% (88–94)	85% (78–89)
<2 mo	31/192	64.3%	90% (86–93)	82% (74–88)

Abbreviations: CI, confidence interval; VE, vaccine effectiveness.

##### Maternal Pertussis Vaccine Effectiveness by Timing of Vaccination

Vaccine effectiveness was also calculated for vaccination at least 4 weeks before delivery, 1–3 weeks before delivery, and within a week of delivery to 2 weeks after delivery (Table 3). For these analyses, cases from week 40 of 2012 were included. Vaccine effectiveness was 91% for infants whose mothers received vaccine at least 4 weeks prior to delivery (95% CI, 88%–94%) and 1–3 weeks prior to delivery (95% CI, 80%–96%). For the small number of infants whose mothers received vaccine up to 1 week before delivery and within 1–2 weeks following delivery, VE declines to 43% (95% CI, −35% to 76%).

**Table 3.** Maternal Pertussis Vaccine Effectiveness Estimates, by Timing of Vaccination

Timing of Vaccination	Cases Vaccinated/Total	Average Matched Coverage	VE (95% CI)
28 d before delivery	31/229	64.1%	91% (88–94)
7–27 d before delivery	4/213	16.2%	91% (80–96)
0–6 d before or 1–13 d after delivery	3/179	2.7%	43% (−35 to 76)

Abbreviations: CI, confidence interval; VE, vaccine effectiveness.

##### Maternal Vaccine Effectiveness Against Infant Death From Pertussis

From the 243 cases, there were a total of 11 deaths in infants, of whom 1 infant had a mother who had been vaccinated at least 1 week before delivery, but <10 days. The average matched coverage was 67.9%. To calculate VE, average matched coverage was applied to the 95% binomial CI on 1/11 (0.2%–31.2%). VE against death was calculated at 95% (95% CI, 79%–100%), consistent with the overall VE against disease of 91%.

##### Maternal Vaccine Effectiveness Against Disease for dTaP5-IPV and dTaP3-IPV

The maternal VE of dT5aP-IPV and dT3aP-IPV did not significantly differ and was calculated at 93% (95% CI, 89%–95%) and 88% (95% CI, 79%–93%), respectively (Table 4).

**Table 4.** Maternal Pertussis Vaccine Effectiveness, by Vaccine Product

Vaccine	Cases Vaccinated/Total	Average Matched Coverage	VE (95% CI)	VE Reducing Coverage by Relative 20% (95% CI)
dT5aP-IPV	20/172	63.1%	93% (89–95)	87% (80–92)
dT3aP-IPV	15/71	69.3%	88% (79–93)	78% (62–88)

Abbreviations: CI, confidence interval; dT3aP, diphtheria–tetanus–3-component acellular pertussis vaccine; dT5aP, diphtheria–tetanus–5-component acellular pertussis vaccine; IPV, inactivated polio vaccine; VE, vaccine effectiveness.

#### Maternal Vaccine Effectiveness in Infants Commencing Primary Infant Series

A total of 73 children had received a childhood vaccine and were born after week 40 of 2012. Of these 73 children, the mothers of 26 had been vaccinated. Of the 73 children, 43 had received 1 dose, 12 had received 2 doses, and 18 had received 3 doses of their primary pertussis vaccines. Onset date was missing in 17 children (11, 2, and 4 of whom had had 1, 2, and 3 doses, respectively).

Estimated VE (Table 5) indicates that maternal vaccine continues to offer protection to children who have received a first primary dose (VE, 82% [95% CI, 65%–91%]). For infants who have received 2 doses, the protection conferred through maternal immunization declines to 69% (95% CI, 8%–90%), and after completion of the primary infant schedule the protection from maternal immunization, which is based on small numbers, declines further, although the point estimate remains above 0%. With these lower effectiveness estimates, the effect of reducing coverage by a relative 20% is greater, with the estimated effectiveness from maternal vaccination declining from 69% to 43% for infants who have received 2 doses of their primary series. Results were similar when only including cases with a known onset date.

**Table 5.** Vaccine Effectiveness of a Maternal Dose at Least 7 Days Before Birth for Infants Commencing Primary Series

Primary Doses	Cases' Mothers Vaccinated/Total	Case Coverage	Average Matched Coverage	VE (95% CI)	VE Reducing Coverage by Relative 20% (95% CI)
Exactly 1 dose	11/43	25.6%	64.3%	82% (65–91)	68% (37–83)
Exactly 2 doses	5/12	41.7%	70.3%	69% (8–90)	43% (−73 to 81)
Exactly 3 doses	10/18	55.6%	64.1%	29% (−112 to 76)	−21% (−242 to 57)
Exactly 1 dose (onset known)	9/32	28.1%	65.5%	81% (57–91)	65% (24–84)
Exactly 2 doses (onset known)	5/10	50.0%	69.2%	56% (−33 to 86)	20% (−156 to 75)
Exactly 3 doses (onset known)	7/14	50.0%	64.4%	46% (−96 to 85)	6% (−216 to 72)

Abbreviations: CI, confidence interval; VE, vaccine effectiveness.

## DISCUSSION

Maternal pertussis vaccination was introduced in the United Kingdom in 2012 in response to the highest number of infant deaths observed for more than a decade. Despite very short time scales for implementation of this outbreak response measure, the program was well accepted, with routine coverage in England close to 60% in the first year of the program. This level of coverage has been sustained in the following 2 years, although coverage from 2 data sources has shown fluctuations during the year with coverage increasing during the winter months. This pattern was particularly evident in 2013 and 2014. One potential explanation is that the increase in coverage coincides with the timing of the seasonal influenza vaccination program for pregnant women. Unlike the maternal pertussis vaccination program, in England there is an active call and recall system for the seasonal influenza vaccination program, and both programs are predominantly delivered in primary care. As a result, women who attend for influenza vaccination can be assessed for their eligibility for pertussis vaccine. While considerable efforts are focusing on improving coverage, up to 40% of the antenatal population in England remain unvaccinated, and this reflects the continuing number of infant deaths since the program was introduced in 2012. This is of particular concern, given that pertussis continues to circulate at heightened levels with the average population incidence for the 3-year period following the introduction of the program being almost 2 times higher than that for the 3-year period preceding 2012. This was observed for all age groups >6 months, although the incidence in infants <3 months of age being targeted by the program has been held at low levels, providing ecological evidence of a program impact. In 2015, an increase in laboratory-confirmed cases was observed across all age groups in England, in line with the usual cyclical peaks every 3–4 years; however, the increased incidence in children aged 5–9 years is of note, as this is the only age group where the incidence in 2015 exceeds the 2012 peak and is the first cohort of children in the United Kingdom who were fully primed and boosted with acellular pertussis vaccines only.

The impact of the maternal program on infant disease is further supported by the assessment of VE. This relied on the rapid enhancement of existing surveillance systems to derive the first estimates of protection from the maternal program in England, 1 year following its introduction. Vaccine effectiveness of the program was calculated at >90% for infants whose mothers received vaccine at least 1 week prior to delivery, using both the screening method and case-control study approaches [10, 11], and this level of protection appears to have been sustained in the 3 years following the introduction of the program despite the change to a another acellular vaccine with a different antigen composition.

The screening method used in this study has the advantage of comparing the vaccination status of cases to a large population, but can only adjust for the potential confounders of maternal age and time period and assumes that vaccination coverage data are representative. The evaluation using the screening method found very similar results to a matched case-control study using many of the same cases where controls were matched to the general practice of the cases [10, 11]. This is consistent with the coverage data used from CPRD being representative and no confounding by general practice or region. Furthermore, we are not aware of other factors likely to have a large confounding effect.

The evidence generated from the evaluation of the maternal program in England has been extremely valuable in informing recommendations on pertussis control strategies by the WHO and national immunization advisory committees [1]. Many countries have recommended pertussis vaccination for pregnant women including the United States, Australia, New Zealand, Belgium, and Spain, although evaluation of their programs with respect to protection against clinical disease is ongoing.

Although evidence of high effectiveness and safety of the maternal pertussis program in England has been extremely encouraging and informed the decision by the JCVI in 2014 to continue the program for a further 5 years, a number of questions still remain. This includes the influence of timing of maternal vaccination on protection against disease in infants. Our analysis suggests that similarly high levels of protection are conferred to infants whose mothers received vaccine at least 4 weeks and 1–4 weeks prior to delivery. The levels of protection decline significantly for the relatively low number of infants whose mothers received vaccine around the time of delivery, and any residual protection that may be conferred is likely to reflect the indirect protection from reduced maternal exposure.

The second question that has been raised is how effective maternal vaccination is against infant deaths. Although deaths have continued to occur since the introduction of the program, these have almost exclusively occurred in infants born to unvaccinated mothers and in the 2 exceptions, maternal vaccination did not occur in the optimal period to confer protection to the infant. The estimated 95% effectiveness against infant deaths adds to the evidence base for the recommendation to offer women pertussis vaccine during pregnancy.

Changes to the vaccine product offered through the program in England in 2014, from the 5-component acellular pertussis vaccine to a 3-component acellular pertussis vaccine (with a higher PT and FHA antigen content), also offered an opportunity to estimate the VE of the 2 products. Our analysis suggests that both the 3- and 5-component vaccines provide high levels of protection for infants <2 months of age and, although the point estimates of effectiveness of the 3-component vaccine is 5% lower than that of the 5-component vaccine, they are not significantly different. A randomized clinical trial in England comparing maternal and infant responses in pregnant women vaccinated with each vaccine is nearing completion and will provide additional valuable information.

Finally, one of the key issues that remains an area of continuing discussion is the impact and clinical significance of blunting of the infants' immune response from maternally derived antibodies. Although immunogenicity studies from North America and England have demonstrated lower antibody responses post–primary series in infants born to mothers who received vaccine during pregnancy, the clinical significance of this blunting effect remains unknown, given the lack of agreed correlates of protection for pertussis. To address this question, the additional protection conferred through maternal immunization for infants who have commenced their primary infant schedule can be estimated. This analysis suggests that high levels of protection are conferred to infants who have received their first dose of the primary series. After the third infant dose, the numbers are small and there is no longer evidence of protection. However, importantly, the estimates are above 0%, which indicates that there is no evidence of greater risk of disease after primary immunization, in those infants whose mothers received vaccine during pregnancy. Although this provides some reassurance, given the low numbers, this question requires ongoing assessment to determine if this continues to be the case. It is important to note that some protective effect may be expected to last beyond the primary vaccination series in the child due to the mother being less likely to transmit. This may mask an interference effect in the direct protection in the child. To assess the direct protection in the child, the VE of the primary doses themselves should be estimated among children whose mothers have been vaccinated; however, the numbers currently are too small to make this assessment.

In the United Kingdom, the pertussis program for pregnant women has been recommended as an outbreak response measure, and a routine recommendation by JCVI requires evidence of cost effectiveness. A recent analysis has indicated that while the cost effectiveness of this program is highly dependent on the future incidence of infant disease, which is uncertain, maternal vaccination is the only certain way of protecting vulnerable infants from birth [20].

In February 2016, the JCVI updated its recommendations on the timing of vaccination during pregnancy, indicating that vaccine can be offered earlier than the 28- to 32-week optimal window that had previously been recommended [21]. This was largely based on data from immunogenicity studies suggesting that vaccinating earlier in pregnancy, from 16 weeks, results in high levels of transplacental transfer of maternal antibodies to the infant [22]. From 1 April 2016, women are recommended to receive pertussis vaccine from 20 weeks (although it may be given from 16 weeks), and it is hoped that widening the window of opportunity for vaccination will help to improve coverage of the program in England and therefore maximize this life saving intervention for young infants.
